# Predictors and clinical features of methotrexate (MTX) therapy for ectopic pregnancy

**DOI:** 10.1186/s12884-020-03350-8

**Published:** 2020-10-29

**Authors:** Jing Zhang, Yu Zhang, Lu Gan, Xiao-ying Liu, Shan-ping Du

**Affiliations:** Shaanxi Provincial People’s Hospital, Xian, 710068 Shaanxi China

**Keywords:** Ectopic pregnancy, MTX therapy, β-hCG

## Abstract

**Background:**

Ectopic pregnancy is a major life- and fertility-threatening women’s health concern. As a result of advances in examination technology, an increasing number of ectopic pregnancies can be diagnosed early and treated with medical methods instead of surgery. The aim of this study was to summarize the clinical features and identify the predictors of success of methotrexate (MTX) treatment of ectopic pregnancy.

**Methods:**

This was a retrospective study of 238 ectopic pregnancies treated with MTX in the Department of Gynecology of Shaanxi Provincial People’s Hospital from January 2017 to December 2017.

**Results:**

Patients were divided into two groups: the successful treatment group (*n* = 166) and the failed treatment group (*n* = 72). The overall success rate of MTX therapy for ectopic pregnancy was 69.75%. The mean initial beta-human chorionic gonadotropin (β-hCG) level was significantly lower in the successful treatment group than in the failed treatment group (2538.08 IU/L versus 3533.17 IU/L, *P* = 0.000). The treatment success rate of the group with an initial β-hCG concentration less than 4000 IU/L was significantly higher than that of the group with an initial β-hCG concentration greater than 4000 IU/L. However, the success rate of the group with an initial β-hCG concentration greater than 4000 IU/L was still relatively high (54.55%). β-hCG levels were significantly increased on the 4th day in the failed treatment group (*P* = 0.000). Compared to the initial β-hCG level, the day-4 β-hCG level increased by more than 8.21%, indicating that the treatment was effective. The diagnostic sensitivity was 88.6%, the specificity was 74.5%, and the area under the receiver operating characteristic (ROC) curve was 0.863 (95% confidence interval (CI): 0.805–0.920).

**Conclusions:**

MTX therapy as a treatment option is safe and effective for asymptomatic, hemodynamically stable patients with ectopic pregnancies who are interested in conservative treatment, regardless of the serum β-hCG level or adnexal mass size. The change in the β-hCG level between the initial day and the 4th day is an effective and early predictive tool for the success of MTX therapy for ectopic pregnancy.

## Background

Ectopic pregnancy is a common acute abdominal disease in obstetrics and gynecology with a reported incidence of approximately 2% and is a common cause of maternal death, accounting for 10% of maternal deaths. In recent years, its incidence has increased due to the increase in the prevalence of pelvic inflammatory diseases and the increased use of assisted reproductive technologies [1–3]. Although traditional salpingectomy is a reliable treatment approach, it involves the loss of the fallopian tubes and the risks inherent in undergoing surgery. With improvements in diagnostic technology, most ectopic pregnancies can be identified early and treated with conservative methods [1]. Medical management not only retains the fallopian tubes but also avoids the risk of pain and high costs associated with surgery. Methotrexate (MTX) has been validated and is widely used for the conservative treatment of ectopic pregnancy. However, the protocol suggests that initial beta-human chorionic gonadotropin (β-hCG) level and the size of the adnexal mass should be below certain cutoff values; for example, in China, this treatment is recommended for hemodynamically stable patients with initial β-hCG levels less than 2000 IU/L and the adnexal mass less than 4 cm in diameter. However, the question of whether some hemodynamically stable patients who wish to avoid surgery and do not meet the above criteria can avoid surgery by undergoing conservative treatment remains unanswered. The follow-up of patients treated with MTX requires the assessment of β-hCG levels on days 0, 4, and 7 after MTX is administered at a dose of 50 mg/m^2^. A 15% decrease in the β-hCG level from day 4 to day 7 is defined as a factor indicating initial success that can be used as a basis for follow-up treatment decisions. However, patients treated with this protocol have to experience anxiety while waiting seven days to obtain an assessment of the effectiveness of treatment. It is important to predict the outcome of this treatment earlier so that we can provide patient recommendations such as surgery or the continuation of follow-up. The β-hCG level on day 4 during the course of treatment might give us some indication of the success of the treatment. These questions remain unanswered. The aim of this study was to investigate the factors predictive of therapeutic success and related factors influencing the success rate of conservative treatment.

## Materials and methods

### General data collection

We conducted a retrospective cohort study of 238 patients with tubal ectopic pregnancies who requested conservative medical treatment in the Department of Gynecology of the Shaanxi Provincial People’s Hospital from January 2017 to December 2017. Ectopic pregnancy was diagnosed by means of a standard set of criteria based on clinical and sonological parameters and serial β-hCG values, including the occurrence of persistent/rising β-hCG values without intrauterine evidence of pregnancy on sonography or histological examination and an adnexal mass or extrauterine gestational sac on sonography. All eligible women who consented to receive MTX treatment for ectopic pregnancy were included. The inclusion criteria were as follows: the patient had an unruptured tubal ectopic pregnancy; the patient had no embryonic cardiac activity;the patient desired to retain her future fertility or had refused surgery (especially if the β-hCG level was < 2000 IU/L or if the adnexal mass was 4 cm or less); and the patient had no contraindications for MTX therapy. The presence of minimal free fluid in the pouch of Douglas was not a contraindication for MTX therapy as long as the patient was hemodynamically stable. Women who strongly desired to retain their reproductive function could receive conservative medical treatment on the basis of informed consent regardless of the initial β-hCG level and the size of the adnexal mass.

The exclusion criteria were acute ruptured ectopic pregnancy, nontubal ectopic pregnancy, conservative treatment drug contraindications and loss to follow-up. Successful treatment was defined as a β-hCG level that was normalized, an adnexal mass that was reduced or that had disappeared on B-mode ultrasound, and symptoms of abdominal discomfort that had eased without surgical intervention. All patients received a single intramuscular injection of MTX calculated to deliver a dose of 50 mg/m^2^. Follow-up assessments were conducted once a week until a cure was achieved. Follow-up included assessments of the patient’s general physical condition and abdominal symptoms; monitoring of chemotherapy side effects; blood β-hCG measurements and ultrasound examinations if necessary to check whether the embryo sac had disappeared; assessments to determine whether pelvic free fluid was reduced; and assessments to determine whether the ectopic pregnancy lesions were reduced.

The β-hCG levels were measured on the initial day, the 4th day and the 7th day after MTX was administered. The clinical outcome was analyzed based on the complete resolution of β-hCG levels, the need for additional doses of MTX or recourse to surgery. If the decrease in the β-hCG level of MTX on the 7th day was less than 15% of that on the 4th day, the patient was switched to surgery or given a second injection of MTX according to the patient’s general situation. The percentage change in the β-hCG level (the β-hCG index) between the initial day and the 4th day was calculated for each case and was defined as the difference in the β-hCG level between the initial day and the 4th day divided by the day-0 value and multiplied by 100 (HCG4-HCG0/HCG0*100).

### Statistical methods

The data were analyzed using the Statistical Product and Service Solutions (SPSS) Statistical Package, Version 23.0 (Chicago, Illinois). General data, including age, weight, height, and other normally distributed data, were analyzed using a Student’s t test. The measurement data (including the β-hCG index and β-hCG change trend) were not normally distributed, so the Mann-Whitney U test and the rank-sum test were adopted. Discrete and categorical variables were analyzed with frequency distributions. Screening for extreme values among quantitative variables was performed using Box-Cox plots and histograms. The best cutoff value for the β-hCG index between the initial day and the 4th day was obtained using the receiver operator characteristic (ROC) curve method.

## Results

### Baseline data

A total of 238 women with ectopic pregnancies were treated with MTX therapy. The maximum age of the patients was 44 years, and the minimum age was 15 years. After MTX treatment, all patients were divided into two groups according to the final treatment outcome: there were 166 patients in the successful treatment group and 72 patients who switched from MTX treatment to surgery in the failed treatment group (25 women underwent surgery before day 4 of MTX). Some patients were treated with antimicrobial drugs if necessary, such as if they presented with fever and an elevated white blood cell count (36 patients in the successful treatment group and 11 patients in the failed treatment group, with no statistically significant difference between the two groups). The follow-up duration was 1–6 months.

**Fig. 1:**
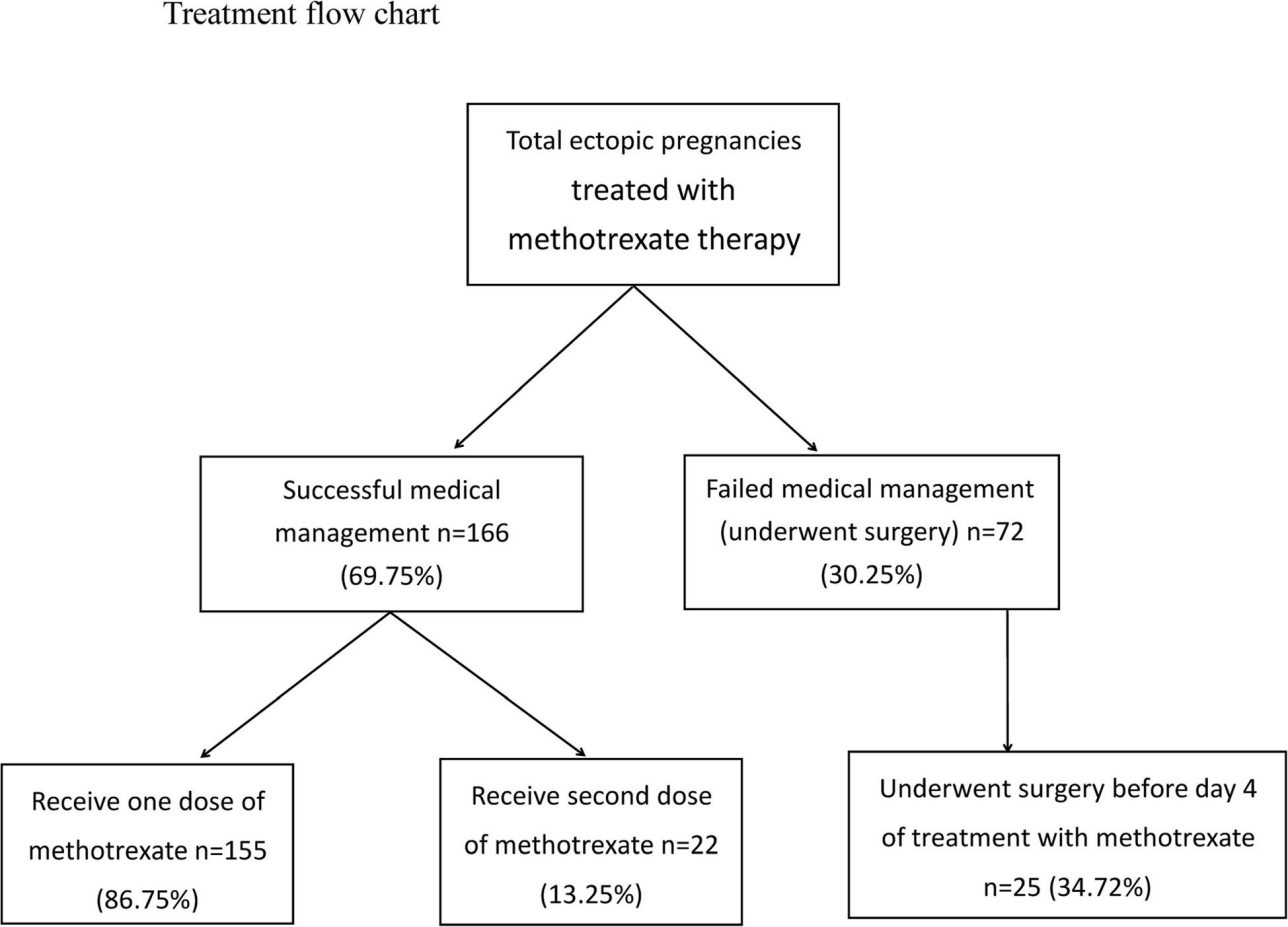
Participants selection flowchart and the number of participants among subgroups of the study population

The overall success rate of MTX therapy was 69.75% (166/238), and 22 patients (13.25%) required a second dose of MTX to achieve complete resolution. There were 72 patients in whom treatment failed (30.25%, 72/238) (Fig. 1). The baseline data for the successful and failed treatment groups are shown in Table 1. There were no statistically significant differences in the demographic profiles of the groups (Table 1).

**Table 1:** Patient characteristics

Variable	Successful (*n* = 166)	Failed (*n* = 72)	*P* value
Age (years)	29.05 ± 4.80	29.85 ± 5.85	0.258^a^
Amenorrhea (days)	47.18 ± 6.79	47.28 ± 6.36	0.833^a^
Parity	1.86 ± 0.96	1.69 ± 1.17	0.510^c^
First pregnancy n (%)	38 (22.89%)	15 (20.83%)	0.726^b^
Body surface area (m^2^)	1.55 ± 0.10	1.54 ± 0.10	0.587^c^
Longest ectopic mass diameter (mm)	2.98 ± 1.25	2.99 ± 1.05	0.932^a^
Initial β-hCG level (IU/L)	2538.08 ± 3107.07	3533.17 ± 1967.80	0.000^c^

(a, mean ± SD and Student’s t-test. b, chi-squared test. c, mean ± SD and Mann-Whitney U test)

### Relationship between β-hCG changes and treatment outcomes

The mean β-hCG levels on day 0, day 4 and day 7 in the successful treatment group and failed treatment group are shown in Table 2. The rank-sum test showed statistically significant differences between the two groups (*P* < 0.001). In total, 92.47% (135/146) of the patients in whom β-hCG levels were decreased on the 4th day were in the successful treatment group. In contrast, in the group of patients in whom the day-4 β-hCG levels had increased, only 56.27% (31/67) were successfully treated with MTX (*P* value = 0.000, chi-squared test).

**Table 2:** β-hCG changes

Variable	Successful (*n* = 166)	Failed (*n* = 72)	Z	P
HCG_0_	2538.08 ± 3107.07	3533.17 ± 1967.80	−4.780	0.000
HCG_4_	1975.83 ± 3443.05	4549.87 ± 3379.32	−6.182	0.000
HCG_7_	1505.50 ± 2044.40	3407.18 ± 3610.90	−3.513	0.000
HCG_0–4_	−0.30 ± 0.48	0.53 ± 0.73	−7.584	0.000
HCG_4–7_	−0.35 ± 0.31	−0.05 ± 0.32	− 0.3981	0.000

The changes in β-hCG levels (hCG_04_) between the initial day and the 4th day were calculated as (HCG_4_-HCG_0_)/HCG_0_. The changes in β-hCG levels (hCG_4–7_) between the 4th day and the 7th day were calculated as (HCG_7_-HCG_4_)/HCG_4_. Negative numbers indicate that the β-hCG levels decreased, and positive numbers indicate that the levels increased (Table 2).

#### The initial β-hCG level and changes in the β-hCG levels between the initial day and day 4 and between day 4 and day 7 predict the outcome of treatment

**Fig. 2:**
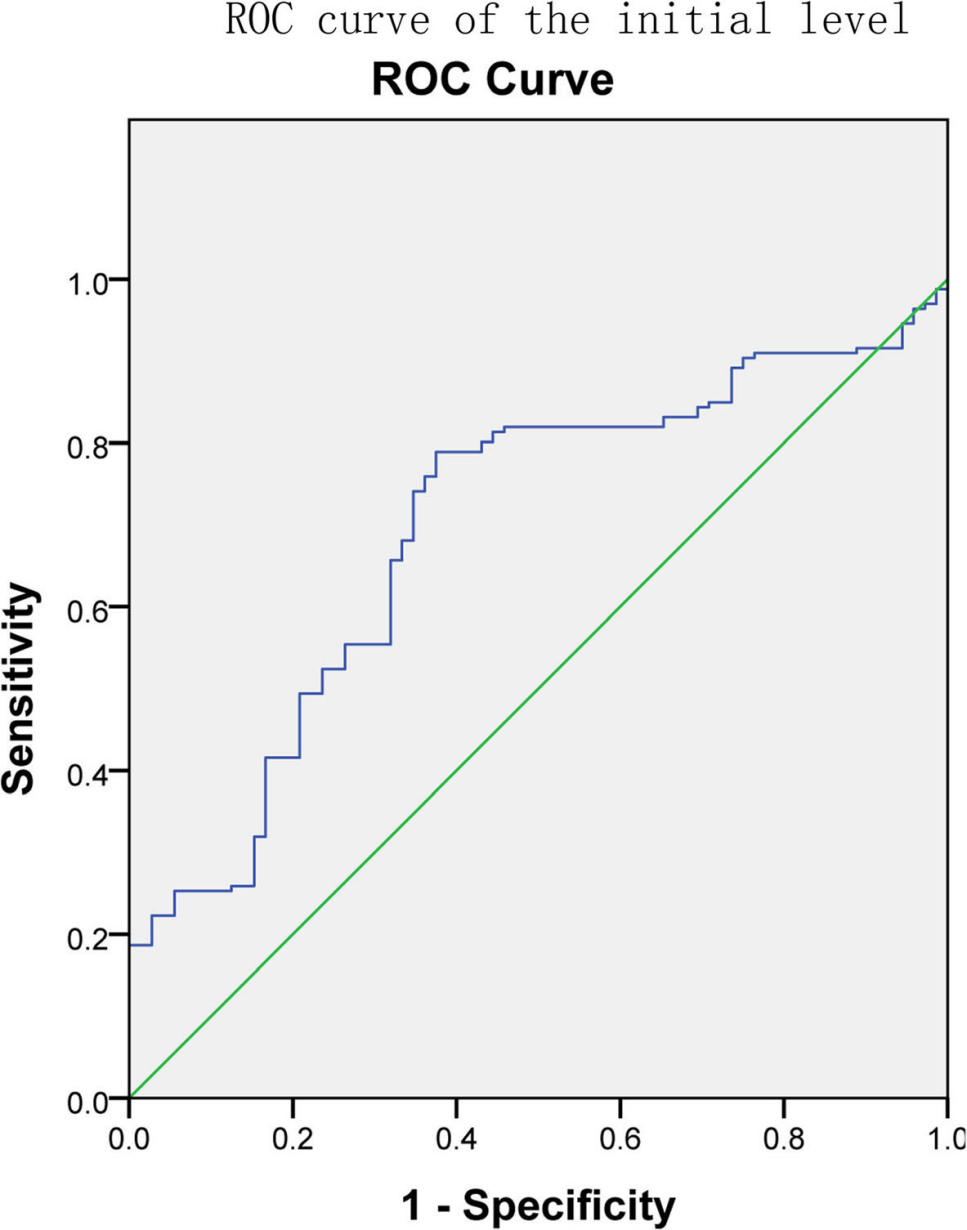
The ROC curves used to evaluate the predictive efficiencies of initial β-hCG level to MTX therapy for ectopic pregnancy. The area under ROC curve was 0.695 (95% CI: 0.624–0.767)

**Fig. 3:**
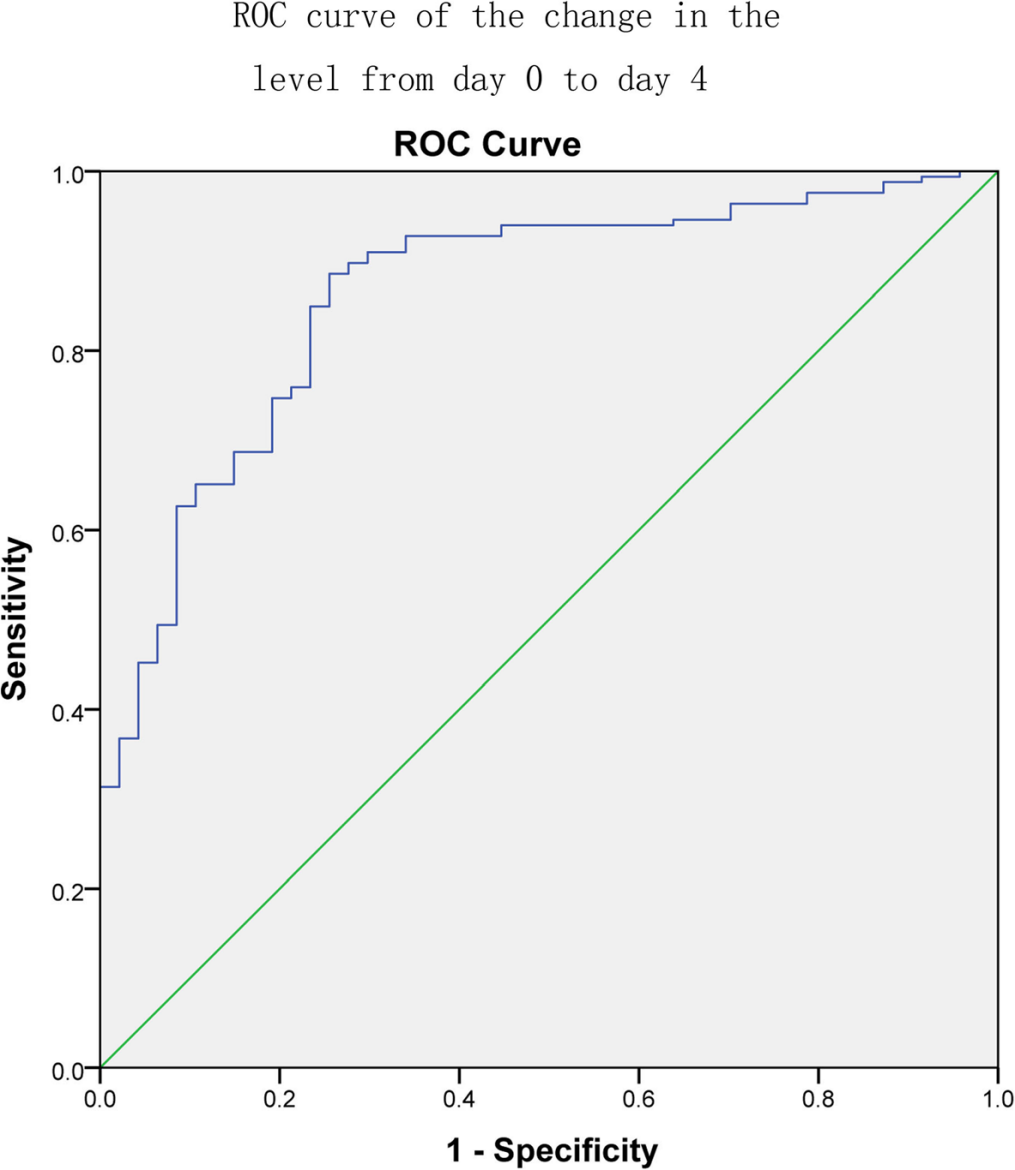
The ROC curves used to evaluate the predictive efficiencies of the change in the level from day 0 to day 4 to MTX therapy for ectopic pregnancy. The area under ROC curve was 0.863 (95% CI: 0.805–0.920)

**Fig. 4:**
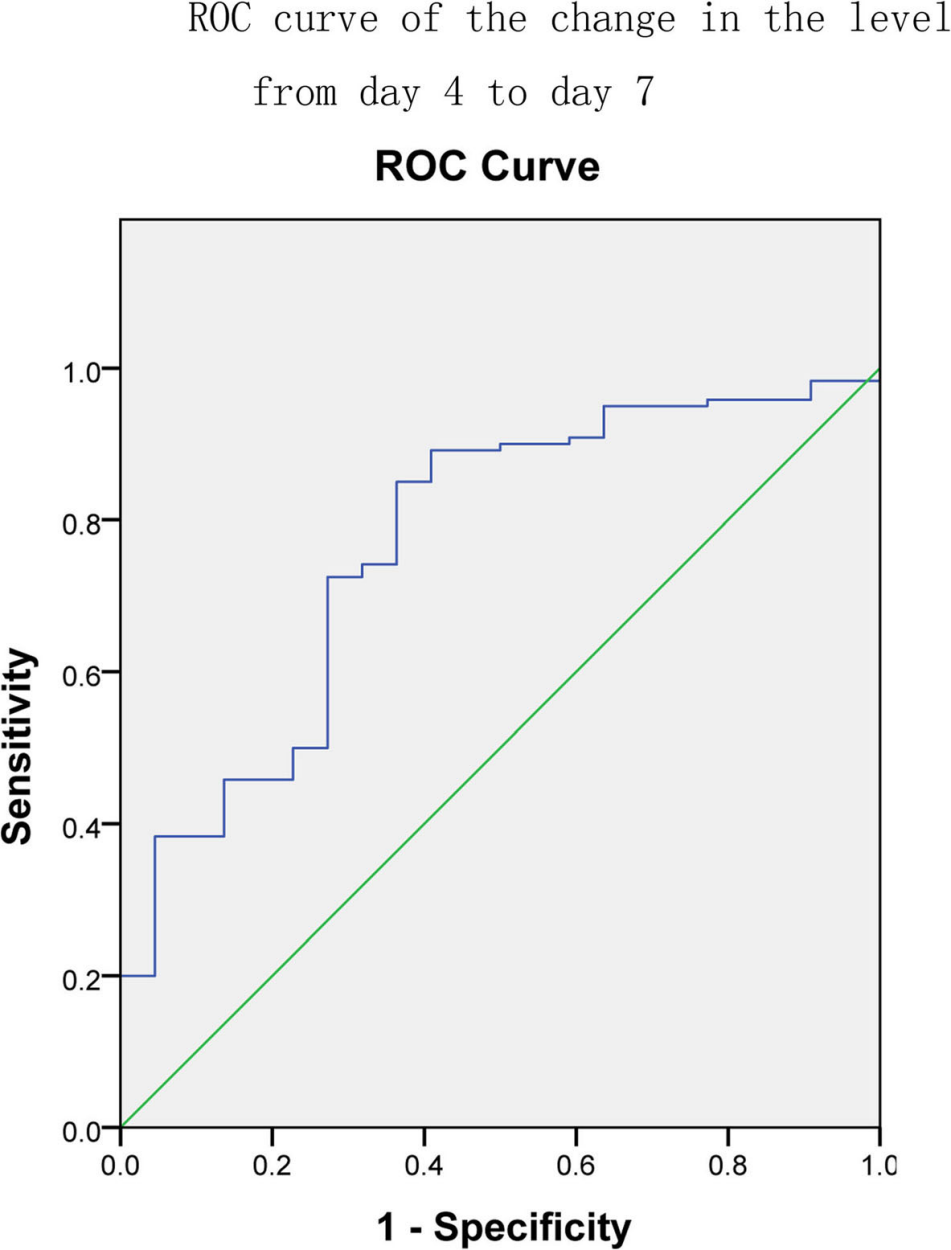
The ROC curves used to evaluate the predictive efficiencies of the change in the level from day 4 to day 7 to MTX therapy for ectopic pregnancy. The area under ROC curve was 0.767 (95% CI: 0.685–0.877)

The areas under the ROC curves (Figs. 2, 3 and 4) for the initial β-hCG level, the change in the level from day 0 to 4 and the change in the level from day 4 to day 7 were 0.695 (95% CI: 0.624–0.767), 0.863 (95% CI: 0.805–0.920) and 0.767 (95% CI: 0.685–0.877), respectively. The diagnostic value of the change from day 0 to day 4 was higher than that of the other two. The best cutoff value for the initial β-hCG level was 3393.78 IU/L, which provided a sensitivity of 78.9% and a specificity of 62.5%. The positive predictive value was 67.78%, and the negative predictive value was 74.76%. The best cutoff value for the change in the β-hCG level from day 0 to day 4 was 0.082 (8.2%). This cutoff value provided a sensitivity of 88.6% and a specificity of 74.5%. The positive predictive value was 77.65%, and the negative predictive value was 86.73%. The best cutoff value for the change in the β-hCG level from day 4 to day 7 was − 0.139 (− 13.9%). This cutoff value provided a sensitivity of 85% and a specificity of 63.6%. The positive predictive value was 70.02%, and the negative predictive value was 80.92%. The various cutoff values for the initial β-hCG level, the change in the level from day 0 to day 4 and the change in the level from day 4 to day 7 are depicted in Tables 3, 4 and 5, respectively.

**Table 3:** Performance of various cut-off levels of HCG_0_

Positive if less than or equal to	Sensitivity	1-Specificity
668.125	0.187	0
723.775	0.223	0.028
758.17	0.253	0.056
806.55	0.259	0.125
962.28	0.319	0.153
1183.585	0.416	0.167
1540.615	0.494	0.208
1801.775	0.524	0.236
1896.08	0.554	0.292
2339.585	0.657	0.319
2679.335	0.681	0.333
3166.525	0.741	0.347
3297.475	0.759	0.361
**3393.775**	**0.789**	**0.375**
3689	0.801	0.431
3855.89	0.813	0.444
3907.83	0.819	0.458
4342.15	0.831	0.653
4369.375	0.843	0.694
4389.52	0.849	0.708
4744.325	0.892	0.736
4808.21	0.904	0.75
5148.365	0.91	0.764
5618.305	0.916	0.889
6706.185	0.946	0.944
7408.48	0.964	0.958
8276.08	0.97	0.972
9105.11	0.988	0.986
24,610.9	1	1

**Table 4:** Performance of various cut-off levels of percentage changes in β-hCG from days 0 to 4 of methotrexate

Positive if less than or equal to	Sensitivity	1-Specificity
−1.980036	.000	.000
−.570668	.313	.000
−.491563	.367	.021
−.381242	.452	.043
−.331703	.494	.064
−.213355	.627	.085
−.181529	.651	.106
−.137062	.687	.149
−.084988	.747	.191
−.057457	.759	.213
.032176	.849	.234
**.082122**	**.886**	**.255**
.155522	.898	.277
.188001	.910	.298
.246691	.928	.340
.355521	.940	.447
.553166	.946	.638
.859267	.964	.702
1.027128	.976	.787
1.261856	.988	.872
1.704043	.994	.915
2.331332	1.000	.957
3.600508	1.000	1.000

**Table 5:** Performance of various cut-off levels of percentage changes in β-hCG from days 4 to 7 of methotrexate

Positive if less than or equal to	Sensitivity	1-Specificity
−0.6	0.2	0
−0.422	0.383	0.045
−0.374	0.458	0.136
−0.35	0.5	0.227
−0.247	0.725	0.273
−0.23	0.742	0.318
**−0.139**	**0.85**	**0.364**
−0.095	0.892	0.409
−0.071	0.9	0.5
−0.034	0.908	0.591
0.121	0.95	0.636
0.223	0.958	0.773
0.476	0.983	0.909
1.989	1	1

The chi-squared test was used to analyze the difference between the treatment success rate of the group with an initial β-hCG concentration greater than 4000 IU/L and that of the group with an initial β-hCG concentration less than 4000 IU/L (*P* = 0.005 < 0.05), and the test indicated a significant difference. However, the success rate for the group with an initial β-hCG concentration greater than 4000 IU/L was still more than 50% (54.55%, Table 6).

**Table 6:** The outcomes of initial β-hCG ranges

hCG	Number of patients	Successful (*n* = 166)	Failed (*n* = 72)	*P* value
≤4000	183	74.32% (136)	25.68% (47)	0.005
> 4000	55	54.55% (30)	45.45% (25)	

### Side effects

Of the 238 patients, 11 patients (4.62%) experienced nausea and vomiting after receiving the medication (similar to after receiving 10 mg of metoclopramide via intramuscular injection), which resolved without intervention, and 5 patients (2.10%) showed slight increases in transaminase levels, which normalized after hepatoprotective drug treatment (456 mg polyene phosphatidylcholine capsules 3 times/day with liver function evaluated every week until it returned to normal); however, the other patients had no obvious adverse reactions.

## Discussion

MTX treatment is safe and effective. According to the statistics in the literature, the success rate of MTX treatment varies from 65 to 95%, with a mean rate of 82%, and the fertility rate with delivery after medical treatment for ectopic pregnancy is 67–80.7%, which is not less than that after conservative and radical surgery [4–7], [8–12]. Meanwhile, MTX does not damage ovarian function or increase the incidence of subsequent adverse pregnancy and birth outcomes in patients [13–15]. An increasing number of patients with unruptured ectopic pregnancies are seeking MTX treatment [16]; however, it is unknown which patients will benefit the most from it. Many studies have tried to identify the factors that influence the success rate before starting treatment with MTX, such as the initial β-hCG concentration, the size of the ectopic mass and a history of previous ectopic pregnancy [17]. Most studies found that only the initial β-hCG level might be a predictor of successful MTX therapy in patients with ectopic pregnancy [8, 9, 18, 19]. The present results were consistent with those of previous studies. The success rate of the group with an initial β-hCG concentration less than 4000 IU/L was significantly higher than that of the group with an initial β-hCG concentration greater than 4000 IU/L. The size of the ectopic mass and a history of previous ectopic pregnancy were independent of the success rate. In contrast, Natale A et al. found no significant difference in initial β-hCG levels between the successful and unsuccessful treatment groups [8]. A retrospective analysis of ectopic pregnancy over a 10-year period published by Alison Richardson found that even when the initial β-hCG level was greater than 3000 IU/L, medical management was still safe and successful, as 85.3% of women in this subgroup avoided surgery [8]. Our results also suggested that the success rate of the group with an initial β-hCG concentration greater than 4000 IU/L was still higher than 50%. Thus, MTX should be more readily considered as a viable treatment option for asymptomatic, hemodynamically stable women with ectopic pregnancies regardless of their initial serum β-hCG levels or adnexal mass sizes, however, they need a longer follow-up duration for repeated doses of MTX and have an increased incidence of emergency surgical intervention.

Although MTX treatment is an effective and safe option for stable patients with ectopic pregnancy, when the traditional diagnostic criterion, a > 15% decrease in hCG between days 4 and 7 after administration, is used, prognostic information can be obtained only on day 7 of treatment. This disadvantage increases patient anxiety and the use of hospital resources and reduces treatment compliance and selectivity. Early predictors have long been a topic of concern among investigators. Obtaining early indicators of successful treatment may reduce the economic burden and psychological stress imposed on the patients, which has the potential to improve patient compliance. The literature reports that there is a transient increase in the β-hCG concentration in 26–60% of women who receive MTX therapy due to the effect of MTX on trophoblast cells [8–12]. This study also found that 28.16% of patients had higher β-hCG levels on the 4th day after MTX treatment, and the mean β-hCG level in the failed treatment group was significantly higher on the 4th day than on the initial day. Many previous studies have shown that changes in the serum β-hCG levels from day 0 to day 4 can provide an earlier indication of MTX treatment success for ectopic pregnancies. Agostini et al. [20] found that a cutoff level of a 20% decrease in HCG levels between days 1 and 4 predicted the success rate with 97% sensitivity. Ustunyurt et al. [11] and Levin Gabriel et al. [21] proposed that a > 22% decrease in β-hCG levels between days 1 and 4 is the best predictor of the treatment success of a single-dose regimen. Girija S. et al. [22] reported a cutoff value of a 10% decrease in β-hCG levels between days 1 and 4 to predict the treatment success rate with a sensitivity of 77% and a specificity of 81%. Lo Wong et al [23] suggested that a 6% drop in serum β-hCG from day 0 to day 4 was the best predictor of treatment success, with a positive predictive value of 91%, which was not inferior to the traditional criterion from day 4 to day 7. In our study, 92.47% of women with a decrease in the serum level of β-hCG from day 0 to day 4 were successfully treated without requiring surgical intervention. Furthermore, based on the ROC curve, a decrease in the β-hCG level of at least 8.2% between the initial day and the 4th day was associated with an 88.6% probability of therapeutic success without further intervention, which was higher than the current criterion of a 15% decrease in the β-hCG level from day 4 to day 7 (76.7%) and the initial β-hCG level (69.5%) to predict treatment success. In addition, in all the previous studies mentioned above, treatment success was defined based on a single dose of MTX, and patients who received two or more doses and/or surgery were classified in the failed treatment group. To the best of our knowledge, the present study is the first trial to classify all patients who did not need surgery in the successful treatment group, including those who received two or more doses of MTX. The common adverse reactions to MTX treatment for ectopic pregnancies, such as stomatitis and nausea, are usually mild and self-limiting. In our study, the incidence of adverse reactions in patients receiving treatment with MTX was only 5.16%, and all of the side effects were mild and reversible, even when the patients received two or more injections.

## Conclusion

In conclusion, MTX therapy for ectopic pregnancy is a safe, effective and superior choice for women with ectopic pregnancies who are asymptomatic and hemodynamically stable regardless of their initial serum β-hCG levels or adnexal mass sizes. The change in the β-hCG level between day 0 and day 4 is a good predictor of the treatment outcome.

## Data Availability

Data are available upon request due to ethical restrictions. Interested researchers may submit requests to Mrs. Jing Zhang for access to sensitive data. Contact: Shaanxi Provincial People’s Hospital, Shaanxi Xian 710068, China. E-mail: jingice24@163.com

## Abbreviations

MTX: Methotrexate
β-hCG: Beta-human chorionic gonadotropin
ROC: Receiver operator characteristic
